# Spatio-temporal patterns of *Synechococcus* oligotypes in Moroccan lagoonal environments

**DOI:** 10.1038/s41598-022-27263-y

**Published:** 2023-01-03

**Authors:** Bouchra Chaouni, Abdellah Idrissi Azami, Sanae Raoui, Saaïd Amzazi, Chakib Nejjari, Fadil Bakkali, El Houssine Zaid, Noureddine Hamamouch, Linda Amaral-Zettler, Hassan Ghazal

**Affiliations:** 1 Laboratory of Plant and Microbial Biotechnology, Biodiversity and Environment, Faculty of Sciences, Mohammed V University in Rabat, 10000 Rabat, Morocco; 2 Josephine Bay Paul Center for Comparative Molecular Biology and Evolution, Marine Biological Laboratory, Woods Hole, MA 02543 USA; 3 Laboratory of Clinical Toxicology, Toxicogenomics and Ecotoxicology, Mohammed VI Center for Research and Innovation, Rabat, Morocco; 4 School of Medicine, Mohammed VI University of Health Sciences, Casablanca, Morocco; 5 Laboratory of Genomics, Bioinformatics and Digital Health, Mohammed VI Center for Research and Innovation, Rabat, Morocco; 6 Laboratory of Human Pathologies Biology, Faculty of Sciences, Mohammed V University in Rabat, 10000 Rabat, Morocco; 7 Present Address: Department of Marine Microbiology and Biogeochemistry, NIOZ Royal Netherlands Institute for Sea Research, P.O. Box 59, 1790 AB Den Burg, The Netherlands; 8 National Center for Scientific and Technical Research (CNRST), Angle Avenues Des FAR et Allal El Fassi, Hay Ryad, B.P. 8027 N U, 10102 Rabat, Morocco

**Keywords:** Microbial genetics, Genetics, Ecology, Microbial ecology

## Abstract

*Synechococcus* are unicellular *cyanobacteria* susceptible to environmental fluctuations and can be used as bioindicators of eutrophication in marine ecosystems. We examined their distribution in two Moroccan lagoons, Marchica on the Mediterranean coast and Oualidia on the Atlantic, in the summers of 2014 and 2015 using 16S rRNA amplicon oligotyping. *Synechococcus* representatives recruited a higher number of reads from the 16S rRNA in Marchica in comparison to Oualidia. We identified 31 *Synechococcus* oligotypes that clustered into 10 clades with different distribution patterns. The *Synechococcus* community was mainly represented by oligotype 1 (clade III) in Marchica. Cooccurring clades IV and I had an important relative abundance in Marchica in the summer of 2014, which is unusual, as these clades are widespread in cold waters. Moreover, Clades VII and subcluster “5.3” formed a sizeable percentage of the *Synechococcus* community in Marchica. Notably, we found low *Synechococcus* sequence counts in the Atlantic Lagoon. These results showed that the relative abundance of *Synechococcus* reads is not constant over space and time and that rare members of the *Synechococcus* community did not follow a consistent pattern. Further studies are required to decipher *Synechococcus* dynamics and the impact of environmental parameters on their spatial and temporal distributions.

## Introduction

*Synechococcus* is an important group of *Cyanobacteria* that contribute to global biogeochemical cycles^1^. They offer an attractive system to explore bacterial taxa relationships, distribution, coexistence, ecology, and evolution^2^. The widespread distribution of this group can be attributed to its high degree of genetic diversity^3^. *Synechococcus* comprises some of the major forms of C*yanobacteria* that inhabit marine and freshwater environments^3^. Although their genetic diversity has been documented for these ecosystems, there is scant knowledge of the biodiversity, abundance, and distribution of this genus in coastal lagoons. The latter environments are highly productive and valued ecosystems while being morphologically and ecologically complex. Coastal lagoons are subject to more variable environmental conditions than the open sea. Due to relative isolation from the sea and their location within a hydrological catchment, these lagoons are more susceptible to changes in physicochemical parameters, leading to increasing salinity, a decrease in nutrient availability and concentration, and light spectral intensity. These factors influence the adaptation strategies of photosynthetic microorganisms, including *Synechococcus* species.

The emergence of next-generation sequencing approaches has granted astonishing insight into microbial biodiversity. Notably, the use of the gold standard gene marker for 16S rRNA now enables microbial diversity assessment across the globe in distinct seasons and locations depending on different environmental conditions^4^. Recently, the use of high-resolution methods, such as oligotyping, has allowed researchers to investigate unexplained diversity within operational taxonomic units and uncover ecologically and biologically distinct taxa^5^. Based on these techniques, seasonal and geographical behavioral patterns of *Synechococcus* strain abundances and distributions have been described in several studies^6–10^. Some studies reported higher abundances in the summer season^6–8^, whereas other studies have shown temporary blooms in spring or summer under eutrophic conditions^9,10^. In both cases, correlations between physicochemical parameters such as temperature, salinity, and nitrate concentration and *Synechococcus* abundances were reported^8,10–12^. Spatial differences were also observed; *Synechococcus* strains were more abundant in coastal waters than in estuaries. Surprisingly, high *Synechococcus* cells abundance was observed even in polar oceans, which are thought to be devoid of them^13^. Twenty *Synechococcus* clades with different patterns of distribution have been described^7^. Indeed, many *Synechococcus* clades, including Clades I and IV, have been observed in temperate or polar waters, as well as coastal and higher latitude regions. The question of consistent co-occurrence (for instance, between Clades I and IV) remains partially unanswered, and the role of environmental parameters remains poorly understood.

*Synechococcus* strains have been classified into three major subclusters (5.1, 5.2, and 5.3) based on 16S rRNA gene phylogeny. Marker genes used to study the diversity of marine *Synechococcus* were essential to describe an important number of subclades, providing more accurate resolution, such as the internally transcribed spacer (ITS), catalase-peroxidase gene (cpeA), nitrate reductase gene (narB), global nitrogen regulator gene (ntcA), ribulose bisphosphate carboxylase large chain gene (rbcL), DNA-directed RNA polymerase subunit beta gene (rpoC1), and especially petB gene coding for cytochrome b6f. This latter gene helped identify more than thirty subclades^3,4^. Furthermore, these markers divided the identified subclusters into more than twenty distinct genetic clades^4^. Marine *Synechococcal* clades are significantly diverse in terms of depth, temperature, and nutrient availability requirements. Clade II is found in tropical offshore environments, with 'hot spots found in the nutrient-rich coastal upwelling of Morocco and causing seasonal blooms in the Red Sea and the Gulf of Aqaba^1^. Clade III is predominantly found in tropical and subtropical warm waters^1^, whereas Clades I and IV occur in nutrient-rich, temperate, and cold environments either nearshore or offshore^1^. Most of these clades (I, II, III, IV, and CRD1) belong to subcluster 5.1. Marine subcluster 5.2 has been observed in nearshore, coastal, and estuarine environments^1^. Different clades may co-occur in similar ecological niches, with reports of as many as six clades found at once^4^. Co-occurrence patterns are observed in coastal waters. Clades II and III co-occurred in the Californian Current during the spring pre-bloom period, while clades I and IV predominated when the bloom itself occurred. Clades V, VI, and X coexist in the Red Sea during transitional periods between mixing and stratification^8^. Abundant *Synechococcus* clades are impacted by limiting factors such as light, nutrient availability, temperature, or viral infections^8^. These factors might also change across seasons and over time scales of environmental changes, leading to clade coexistence^7^. When less abundant, they may persist at low but stable levels^14–16^ and serve as reservoirs of genetic diversity^8^.

In this study, we used 16S rRNA amplicon oligotyping to investigate and compare *Synechococcus* population diversity and co-occurrence patterns in two Moroccan lagoons, Marchica and Oualidia, during the summers of 2014 and 2015. We hypothesized that the distribution and cooccurrence of *Synechococcus* oligotypes follow spatiotemporal patterns in lagoonal environments.

## Results

### Detection of *Synechococcus* oligotypes in environmental samples

**Figure 1:**
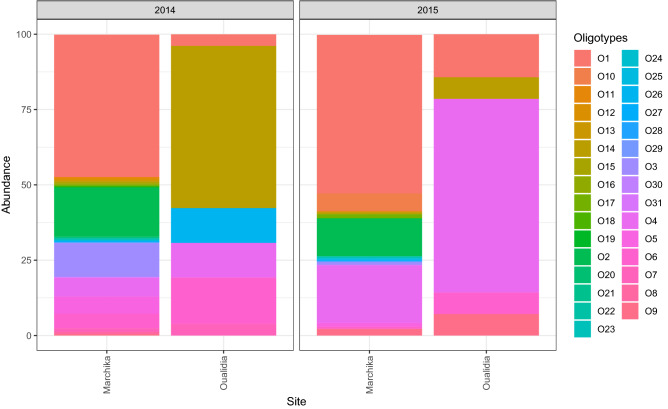
Cross comparison of 31 *Synechococcus* oligotypes in Marchica and Oualidia lagoons in 2014 and 2015.

A total of 535,138 16S rRNA amplicon sequence reads were generated from the microbial populations of four samples collected at two stations on the summer solstice, 21st June in 2014 and 2015. The relative *Synechococcus* read number compared to the total microbial community varied between both sampling sites during both timepoints (Fig. 1 and Table 1).

**Table 1:** V4–V5 Oligotype observation matrix.

Oligotype	Oligotype ID	Sites (abundance count)				Sites (abundance percent)			
		OSD2414	OSD 2415	OSD9114	OSD9115	OSD2414	OSD2415	OSD9114	OSD9115
TTAATCT	O1	7290	878	1	2	47.19	52.66	3.84	14.28
GACTCTC	O2	2509	208	0	0	16.24	12.47	0	0
TTCATCT	O3	1727	15	0	0	11.18	0.89	0	0
TTAAGCT	O4	974	319	3	9	6.30	19.13	11.53	64.28
GACTCCT	O5	874	20	0	0	5.65	1.19	0	0
TTAATTC	O6	750	13	4	1	4.85	0.77	15.38	7.14
TTCATTC	O7	177	0	1	0	1.14	0	3.84	0
ATAATTC	O8	119	0	0	0	0.77	0	0	0
TTAATTT	O9	76	37	0	1	0.49	2.21	0	7.14
GTCTCCT	O10	16	98	0	0	0.10	5.87	0	0
TTCTCTC	O11	86	1	0	0	0.55	0.05	0	0
TTAAGTC	O12	74	5	0	0	0.47	0.29	0	0
AGAATTC	O13	70	0	0	0	0.45	0	0	0
AAAATTC	O14	44	11	14	1	0.28	0.65	53.84	7.14
GACATCT	O15	67	1	0	0	0.43	0.05	0	0
TTAACTC	O16	65	0	0	0	0.42	0	0	0
AGCATCT	O17	41	21	0	0	0.26	1.25	0	0
ATAATCT	O18	53	0	0	0	0.34	0	0	0
TTAACCT	O19	46	3	0	0	0.29	0.17	0	0
TTATCTC	O20	45	0	0	0	0.29	0	0	0
TAAATCT	O21	43	2	0	0	0.27	0.11	0	0
TTCTCCT	O22	43	1	0	0	0.27	0.05	0	0
TTACTCT	O23	36	8	0	0	0.23	0.47	0	0
TTGATCT	O24	37	3	0	0	0.23	0.17	0	0
TTAGTCT	O25	29	5	0	0	0.18	0.29	0	0
TAAATTC	O26	24	7	3	0	0.15	0.41	11.53	0
GACTTCT	O27	29	1	0	0	0.18	0.05	0	0
TTAATCC	O28	26	3	0	0	0.16	0.17	0	0
TCAATCT	O29	25	4	0	0	0.16	0.23	0	0
GAAATCT	O30	29	0	0	0	0.18	0	0	0
CTAATCT	O31	23	3	0	0	0.14	0.17	0	0

*OSD2414: Marchica Lagoon 2014 sampling; *OSD2415: Marchica Lagoon 2014 sampling.

*OSD9114: Oualidia lagoon 2014 sampling; *OSD9115: Oualidia lagoon 2015 sampling.

**Figure 2:**
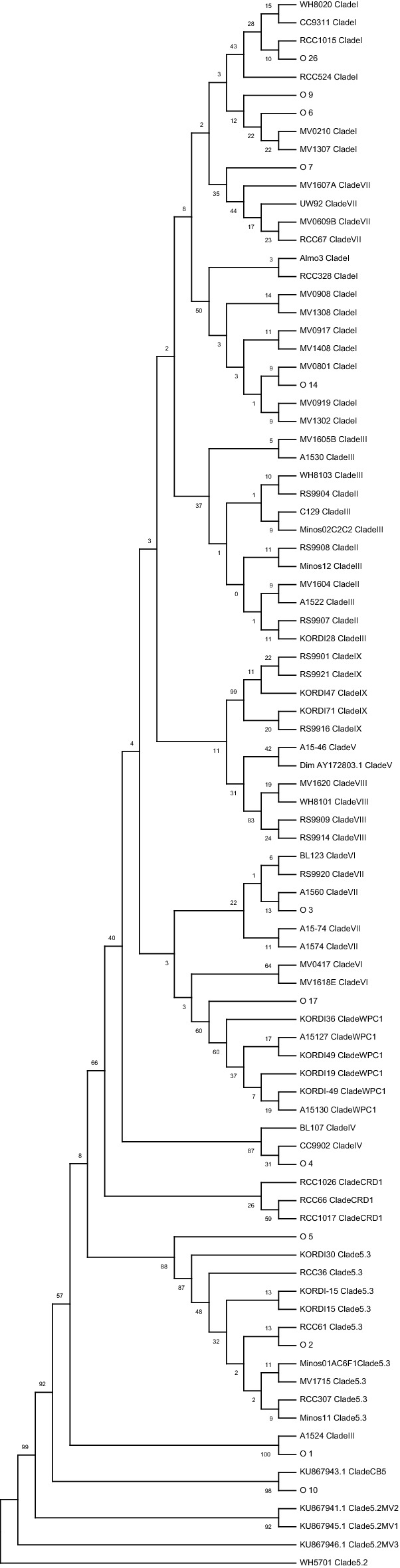
Phylogenetic tree constructed from V4–V5 16S rRNA gene amplicon sequence representatives of *Synechococcus* clade and representative sequences for the most abundant oligotypes, both aligned using Muscle (version 3.8.4,^17^). Shown is a neighbor-joining rooted tree generated using the Geneious Prime 2021.1 software package (Biomatters Ltd, Auckland, New Zealand).

We identified 31 *Synechococcus* oligotypes, which was equivalent to 95% of all *Synechococcus* reads analyzed. The most abundant representative *Synechococcus* reads were used for downstream analyses depending on the sampling location and date. Our phylogeny confirmed that *Synechococcus* strains are classified into ten different clades based on representative V4–V5 16S rRNA sequences (Fig. 2).

The identified clades were separated into three distinct subclusters (5.1, 5.3 and 5.2). Ten oligotypes belonged to clade III (subclade 5.1A) (O1, O15, O19, O21, O23, O24, O25, O29, O30, O31), six belonged to clade I (subclade 5.1B) (O6, O8, O9, O14, O16, O26), another six belonged to Clade 5.3 (O2, O5, O11, O20, O22, O27), two belonged to clade IV (subclade 5.1A) (O4, O12), another two belonged to clade VII (subclade 5.1B) (O3, O7), and only one oligotype belonged to clades II (subclade 5.1A) (O28), CB5 (subclade 5.2) (O10), WPC1 (subclade 5.1) (O17), VIII (subclade 5.1B)(O18), and IX (subclade 5.1B) (O13) (Table S1).

The eight abundant oligotypes in our dataset (i.e., represented by > 100 reads) shared more than 95% V4–V5 sequence similarity with each other (Table 2).

**Table 2:** Percent sequence similarity between V4 and V5 representative sequences for each oligotype.

	O1	O2	O3	O4	O5	O6	O7	O8
O1	–	96.07	99.44	99.44	96.63	99.44	98.88	99.72
O2		–	96.26	95.99	99.47	96.79	96.79	96.52
O3			–	98.93	96.79	98.93	99.47	99.2
O4				–	96.52	98.93	98.4	99.2
O5					–	96.26	96.26	96.52
O6						–	99.47	99.73
O7							–	99.2
O8								–

*Synechococcus* comprised a higher fraction of the microbial population and showed higher relative abundances in the summer of 2014 in Marchica than in 2015. We placed the eight abundant oligotypes within a phylogenetic tree that included known *Synechococcus* strains (Fig. 2). Table 1 shows higher read counts of *Synechococcus* oligotypes in the Marchica Lagoon (n = 15,447), (n = 1,667) in the summers of 2014 and 2015, respectively, compared to Oualidia (n = 26), (n = 14) during both the summers of 2014 and 2015.

Interestingly, Oligotype 1 was strongly represented in both sampling lagoons (Table 1), where it comprised a larger segment of the overall *Synechococcus* community. In contrast to the 2014 summer community in Marchica, a few *Synechococcus* oligotypes decreased in 2015; for instance, O7 changed from 177 *Synechococcus* reads to not detected, and O8 changed from 119 *Synechococcus* reads to not detected. In Oualidia, we noticed the presence of clades III (O1), IV (O4), I (O6, O14, O26), and VII (O7) in 2014 in contrast with 2015, where clade VII was absent and only 5 *Synechococcus* oligotypes were identified: O1, O4, O6, O9 and O14.

### Distribution of *Synechococcus* oligotypes

**Figure 3:**
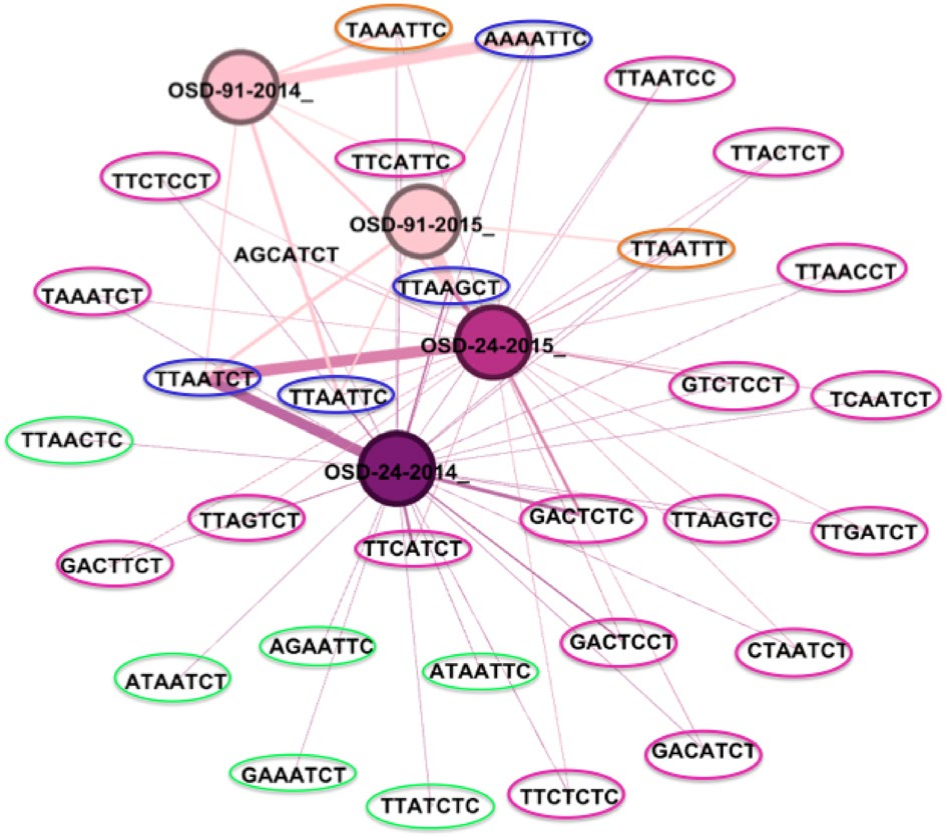
A network analysis of the oligotypes present at each sample. Each dot indicates an oligotype present in at least one sample, and each edge on the network connects an oligotype to one or more samples. Colored circles represent oligotypes present in only one, two, three, or all samples.

Although some oligotype distribution patterns within each sampling site clearly displayed some differences over both space and time, many oligotypes were shared as well. Network analysis allowed visualization of the specificity of the oligotypes and how they were distributed in Mediterranean Marchica and Atlantic Oualidia lagoons and further investigation of which factors influenced this distribution (Fig. 3).

We identified oligotypes that were either found in one collected sample, shared by two samples, or present in all samples (Fig. 3). Oligotypes in OSD24-2014 accounted for the largest fraction (25/31), whereas oligotypes shared by the four samples made up a small portion of the total number (4/31). Most oligotypes were shared between both 2014 and 2015 samples of Mediterranean Marchica, in addition to some overlap with Atlantic Oualidia. Among all oligotypes, six were exclusively found in Marchica in 2014 (Fig. 3). Furthermore, the distribution of cooccurring oligotypes was clearly different in Marchica and Oualidia during the summers of 2014 and 2015. Cooccurring *Synechococcus* oligotypes tended to be well connected to each other (Table 2), showing strong correlations (e.g., O4 (clade IV) and O6 (clade I), = 0.98, O4 (clade IV) and O8 (clade I), = 0.99).

### Environmental variables influencing oligotypes diversity

Following principle component analysis (PCA) of oligotype relative abundance, we observed that physicochemical factors in the lagoons correlated with oligotype co-occurrence patterns. The first principle component (PC1) captured 46% of the variance in oligotype relative abundance and discriminated oligotypes according to nitrate. The second principle component explained an additional 27% of the variation and discriminated oligotypes according to salinity and temperature.

The composition shift seen during the summer was supported by the statistical connections between dominating PCs and environmental variables. Notably, identified oligotypes in Marchica (O2, O3, O4, O5, O6, O7, O9, O10, O14, O15, O16, O17, O26) in 2014 and (O1, O2, O4, O5, O6, O9, O10, O14, O15, O16, O17, O26) in 2015 correlated with higher temperatures and salinities (26 Celcius, 27 Celcius, 35 ppt, 35 ppt, respectively), when oligotypes in Oualidia (O1, O4, O6, O7, O14, O26) in 2014 and (O1, O4, O6, O9, O14) in 2015 correlated with lower ones (21 Celcius, 20 Celcius, 27ppt, 29ppt respectively). Both oligotypes found in Marchica and Oualidia in 2014 correlated with a higher nitrate concentration (12 mg/l, 10 mg/l, respectively). However, those observed in 2015 correlated with a lower nitrate concentration (4 mg/l, 2 mg/l). Furthermore, Oligotype O1 in 2014 was spatially isolated, not affiliated with principal component.

## Discussion

In a previous study^18^, we used bioinformatics tools to analyze the metagenome and the amplicon 16S sequences to gain an insight into microbial diversity in Moroccan lagoons, namely Marchica and Oualidia. 16S rRNA gene classification revealed a high percentage of bacteria in both lagoons. On average, bacteria accounted for 90% of the total prokaryotes in Marchica and ~ 70% in Oualidia. The five phyla that were the most abundant in both lagoons, Marchica and Oualidia, respectively, were Proteobacteria (53.62%, 29.18%), Bacteroidetes (16.46%, 43.49%), Cyanobacteria (0.53%, 34.35%), Verrucomicrobia (1.75%, 15.82%), and Actinobacteria (7.42%, 13.98%). At the genus level, we found that the highest assigned hits were attributed to Synechococcus, which was highly abundant in Marchica (32%) compared to Oualidia (0.07%) in 2014. This amount dropped to 22% in Marchica and 0.04% in Oualidia in 2015. Hence, in this study we performed the analysis of the *Synechococcus* genus community using oligotyping to investigate their dynamics and understand their co-occurrence and covariation in space and time within fragile ecosystems such as lagoons.

We may divide our results into two emerging *Synechococcus* communities: one dominated in 2014 and the other was less present in 2015, each composed of different cooccurring *Synechococcus* oligotypes. The abundant *Synechococcus* community in Marchica in 2014 consisted of clades I, 5.3, III, IV, and VII. These clades are typically found in either warmer or more oligotrophic environments^19,20^. This result is in accordance with Marchica's environmental characteristics; it is an oligotrophic ecosystem with high primary production and warmer water in summer^21^. The community included clades CB5 and WPC1 in Marchica 2014 and 2015 when the number of *Synechococcus* reads was lower. Strains belonging to the CB5 clade lack phycourobilin (PUB), contain one motile strain^22,23^**,** are present in temperate coastal waters and are prevalent in polar/subpolar waters^24–26^. WPC1 strains are observed in open-ocean and near-shore waters^1,24,27^. Clades IV and I usually co-occur and are more prevalent in cold coastal waters^19,28–30^. Interestingly, Clade III was prominent in Marchica. This clade is known to be motile and restricted to warm, oligotrophic water^19,20,30^**.** Although at a smaller read number, clade III was also observed in Oualidia, where the temperature is cooler compared to Marchica. Furthermore, we found that clade III growth has been shown to be severely affected at low temperatures^30^**.** Moreover, representatives of both clades I and IV were present in Oualidia in both the summers of 2014 and 2015. Some *Synechococcus* strains, which are known to prefer cooler water temperatures and salinities, were in higher relative abundance in the waters of Marchica. This result agrees with a previous study showing that *Synechococcus* isolates of clades I and IV exhibited temperature preferences^31^. Their growth rates were marginally lower at low temperatures in strains from clades I and IV, which were dominant in temperate regions.

Nitrate levels are typically low or undetectable in these lagoons, which allows the persistence of clades that would not typically thrive in coastal waters at other times of the year. In 2014, the nitrate concentration was higher than the average of 10 mg/l, which could be due to increased agricultural activities and wastewater treatment plant effluent^21^. The decreasing nitrate concentration in Marchica in 2015 could be explained by the newly installed inlet in 2010, which was designed to improve water exchange with the open sea and reduce the amount of suspended matter^21^. Temperature and salinity have a large effect on nitrate in marine ecosystems^32^; the highest nitrate degradation rates were observed at 35 °C and at increasing salinity rates. Therefore, we expected to see correlations between salinity, temperature and nitrate concentrations. Interestingly, clades CB5 in Marchica and IV in Oualidia increased in relative abundance in summer 2015 compared to 2014, when the nitrate concentration decreased. Moreover, the *Synechococcus* microbial community diversity and density are variables depending on the variations in the physical and chemical parameters. These parameters are strongly influenced by the marine waters passing through the artificial inlets, which have an impact on the internal hydrodynamics of both lagoons and hence the distribution and co-occurrence of *Synechococcus* strains. In addition, anthropogenic activities also have a great influence on *Synechococcales* population growth and interactions with their viruses^33,34^.

This study revealed some differences between Marchica and Oualidia in identified *Synechococcus* clades. The Marchica lagoon showed more heterogeneity (clades I, II, III, IV, VII, VIII, 5.3, WPC1, CB5, and IX) than the Oualidia lagoon, where fewer clades were identified (I, III, IV, and VII). There was a clear variation in the pattern of correlation between oligotypes of the same or different clades for both the 2014 and 2015 samplings. Furthermore, we observed complex patterns of co-occurrence among oligotypes; in 2014 (clades I, III, IV, 5.3, VII), and in 2015, we found clades CB5 and WPC1. In Oualidia, values decreased in comparison to Marchica in both 2014 and 2015 summer samplings, following a pattern of co-occurrence, especially for both clades I and IV in both sampling years. Many studies have shown that the relative proportions of cooccurring *Synechococcus* populations to each other at the clade and subclade levels vary in space and time based on environmental factors such as seasonal temperature fluctuations, nutrient availability and upwelling, circulation patterns, and abundance of other phytoplankton^8^.

We presume that the greater variability in oligotype co-occurrence behavior observed in Marchica Lagoon, especially in the summer of 2014, could be due to the higher abundance and diversity of *Synechococcus* oligotypes, physico-chemical parameter fluctuations or rehabilitation of the lagoon.

**Figure 4:**
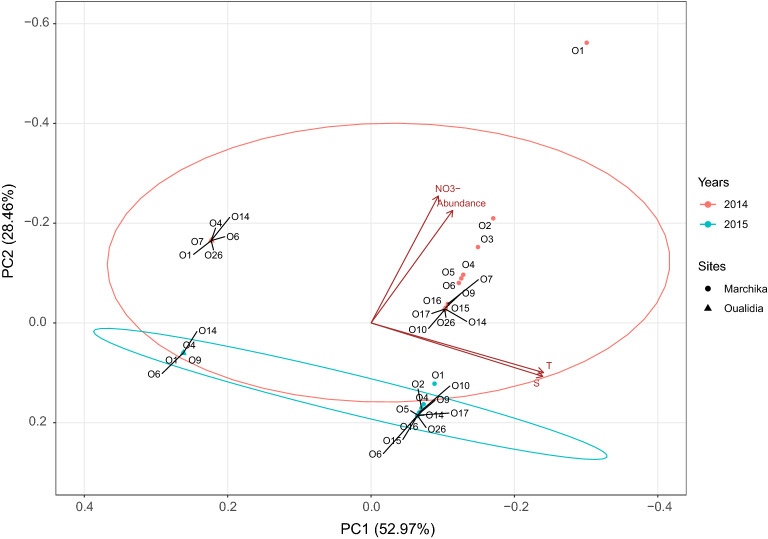
Principle component analysis of *Synechococcus* oligotype relative abundance. The plot is generated using the relative abundance of each oligotype, *T* temperature, *S* Salinity, and *NO*^*3−*^ Nitrate. Each point represents an oligotype. Colors represent the year of sampling; red for 2014 and blue for 2015. The shape of point indicates the sampling site; rounded points refer to Marchica lagoon, and triangles refer to Oualidia. Circles represent the normal distribution of oligotypes; the red circle refers to 2014, and the blue one refers to 2015.

Less abundant oligotypes could also be considered potential bioindicators of *Synechococcus* genetic diversity. Their seasonal occurrence might contribute to changing ecological and biogeochemical characteristics of the marine environment^35^. The *Synechococcus* relative abundance count revealed that the Marchica *Synechococcus* community included the least abundant oligotypes in 2015. For instance, O7 and O8 were detected in 2014 and were absent in 2015 (Table 1). It is unclear which factors served to constrain the relative abundances of these least present oligotypes, but temperature and salinity could have an impact on their distribution in Marchica (Fig. 4) and the opposite for Oualidia, which are cooler-temperature adapted ones. We noticed that the relative abundance of cooccurring *Synechococcus* was not constant. For instance, oligotype 4 belonging to Clade IV showed higher values in summer 2014 (974 reads) in Marchica compared to summer 2015 (319 reads), and the opposite was observed in Oualidia, with a lower abundance compared to Marchica. Increased values of cooccurring clade I oligotypes (14, 26, and 6) were detected in the summer of 2014 in both lagoons.

In comparing our results with a study from Little Sippewissett Marsh (LSM)^8^ that used oligotyping to investigate the distribution of the genus *Synechococcus* in space and time sequencing the V4-V6 hypervariable region of the 16S rRNA gene, we found 31 oligotypes, while they identified 12. In both studies, the proportion of *Synechococcus* oligotypes increased in summer and in coastal waters compared to estuaries. In addition, Clades I and IV were more abundant in saline conditions, such as Marchica Lagoon. However, these clades were found in greater relative abundances at cold temperatures, in contrast to our study, where they were identified in Marchica's warm waters. Moreover, clade CB5 tended to be prominent at relatively warm temperatures (17–20 °C)^6^. In our work, it was not prevalent either in cooler or warmer water. Notably, the relative abundance of rare oligotypes was higher in warm hypersaline estuary waters^8,18^, while in our case study, they occurred in cooler moderately saline Oualidia waters.

The dominance of a certain clade could have many different ecological ramifications, especially as the clades can be incredibly diverse in their growth, loss, nutrient utilization and other attributes. The dominant clade's growth and loss patterns will set the stage for the population dynamics. For instance, if the dominant clade only blooms in a given environmental factor such as temperature, light, or salinity, it will then affect the timing of blooms, and follow-on the effects of subsequent grazing, lysis or even biogeochemical cycling. Even if the population is diverse, the dynamics as a whole will be a composite response of each individual clade's ecophysiology, making it important to understand their composition and how it changes over space and time.

While the rpoC1 gene is a higher resolution diversity marker^36^, 16S amplicon data can be used for exploring the entire bacterial assemblage including *Synechococcus* clade designations via oligotyping^35^. The latter has a great advantage in answering unexplained diversity contained in taxa using 16S rRNA gene sequences. Nevertheless, it has some limitations, as it acts optimally only when performed on taxa that are closely related. Regarding distantly related taxa, the high number of increased-entropy locations makes the supervision steps difficult. In addition, although oligotyping does not rely on clustering conditions or availability of existing reads within reference databases, it demands preliminary operational taxonomic unit clustering to find closely related species appropriate for the analysis. This method is under continuous improvement to better exploit the information within subtle variations in 16S rRNA gene sequences^5^.

In conclusion, we explored the patterns of *Synechococcus* diversity in space and time using an oligotyping approach to examine these populations in lagoon waters of Mediterranean Marchica and Atlantic Oualidia, in Morocco. Patterns that have been observed at the clade and subclade levels, such as *Synechococcus*, relative abundance and the co-occurrence of groups from different clades, were shown to occur among oligotypes. The Marchica Lagoon showed a heterogeneous *Synechococcus* diversity compared to Oualidia in summer 2014. Thirty-one *Synechococcus* oligotypes were identified. Two distinct communities emerged in the 2014 and 2015 summer samplings, abundant and rare *Synechococcus* species, each comprising cooccurring *Synechococcus* oligotypes from different clades. Network analysis showed that six oligotypes were exclusive to Marchica Lagoon. The identified clades I, III, IV, VII, and 5.3 in Marchica were in accordance with its environmental characteristics. In addition, the relative abundance of some cooccurring *Synechococcus* strains was not constant over time and space (e.g., clades I and IV). Using gene oligotyping, we illustrated some of the challenges associated with the identification of novel *Synechococcus* strains or studied their co-occurrence in space and time. Oligotyping has been instrumental in discriminating closely related *Synechococcus* strains. However, this study leaves open questions about how samples differ by location and whether locations differ from year to year. Do cooccurring oligotypes interact with each other and to what extent do they correlate with physicochemical parameters? What triggers the coexistence of clades I and IV with clade III in warm water or 5.3 with VII, which do not know much about. Finally, how do relative abundances change over seasons. Hence, future work needs to consider additional stations and seasons to provide better statistical support for our findings and to better understand their correlation with physical and chemical environmental parameters. Other factors were not considered in this study, such as nutrient availability, chlorophyll, irradiance, viral lysis, and greater sequencing depth, which could also influence the observed seasonal dynamics.

## Methods

### Sampling and sequencing

**Figure 5:**
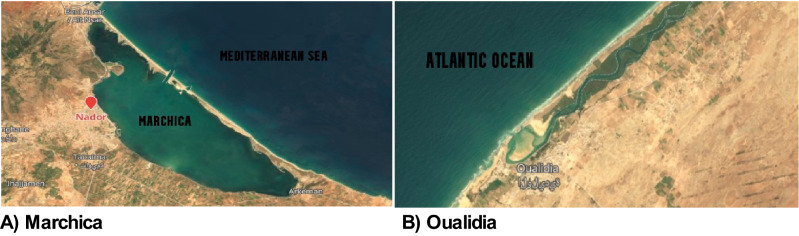
Sampling locations: (**A**) Marchica Lagoon (Image©2022Google) and (**B**) Oualidia Lagoon (Image GeoEye from Google Earth, 2014).

Samples were collected from Marchica Lagoon (N 35.11562, W 2.52803) and Oualidia Lagoon (N 32.74675, W 9.036667) on June 21^st^, 2014, and 2015, boreal summer solstice, as part of the Ocean Sampling Day (OSD) campaign (Fig. 5). Approximately 20 L was collected using a 10% acid-washed bucket and then sequentially filtered onto a 0.22 μm pore size Sterivex and frozen at − 80 °C until DNA extraction. Metadata (temperature, salinity, and nitrate) were measured and uploaded into https://github.com/MicroB3-IS/osd-analysis/wiki/Guide-to-OSD-2014-dataaccessec on 1 December 2021.

DNA was extracted using the Power Water isolation kit (MoBio, Carlsbad, CA, USA) following the manufacturer’s instructions. Amplification of the 16S rRNA gene was performed using the primer pair, designated: 515F-Y (5’-GTGYCAGCMGCCGCGGTAA-3’) and 926R (5’-CCGYCAATTYMTTTRAGTTT-3’)^37^. The Illumina libraries were prepared using the NuGEN Ovation Rapid DR Multiplex System 1–96. Amplicon gene sequencing (2 Å ~ 250 paired ends) was performed with Illumina MiSeq using V3 chemistry. Samples were sequenced in eight MiSeq runs (2 × 300 bp), which generated 2 × 40,000 amplicon reads per sample.

### Data processing

Raw sequencing reads were preprocessed as described in the OSD workflow (github.com/MicroB3-IS/osd-analysis/wiki/Sequence-Data-Preprocessing_accessed on December 1, 2021), which produced "workable" amplicon fasta files. We used VAMPS^38^ to process 16S rRNA gene sequences, where taxonomy assignment was performed using Global Alignment for Sequence Taxonomy (GAST)^39^ and the SILVA rRNA gene reference database^40^. The obtained files include the reference ID, the taxonomy assigned, and the source of the taxonomy.

### Oligotyping

For *Synechococcus* investigation, we used 16S rRNA gene oligotyping as described in^5^. This method is based on a supervised algorithm that identifies microdiversity using 16S rRNA gene sequences. Oligotyping is unlike regular taxonomic classification based on available reference databases available sequences or cluster analysis based on the selection of the similarity threshold. This technique tackles the taxonomic resolution limitation by finding the most information-rich nucleotide positions (i.e., oligotypes). Sequences identified as *Synechococcus were extracted* from the Vamps database. We aligned *Synechococcus* reads using PyNAST^41^. Of the 22,387 sequences identified as *Synechococcus*, 17,941 remained after quality filtration and Pynast alignment. The mean length of *Synechococcus* reads was 254 bp. Next, we removed the uninformative gaps in the resulting aligned sequences using the “o-trim-uninformative-columns-from-alignment” script. Subsequently, we calculated the entropy of each nucleotide position within the oligotype package. After the initial calculation of Shannon entropy using the “analyze-entropy” script, we ran 16S rRNA oligotyping for the S*ynechococcus* genus until each oligotype had converged. Uninformative nucleotide positions were excluded. Seven nucleotide positions were used in total to define each oligotype, and to minimize the impact of sequencing errors on oligotyping results, we used a “minimum substantive abundance” criterion (M) of 5; thus, an oligotype was not included if the most common sequence for that type occurred less than five times. To reduce the noise, each oligotype was required to appear in at least one sample but was not required to comprise a certain percentage of reads or represent a minimum number of reads in all samples combined. We removed any oligotypes that did not meet these criteria from the analysis. The final number of quality-controlled oligotypes revealed by the analysis was 31 and represented 95% of the total *Synechococcus* reads. For each oligotype, the oligotyping pipeline chose the most abundant read as the representative sequence to be used for downstream analyses. Upon completion of oligotyping analysis, the resulting “observation matrices” are concatenated to generate a single “observation matrix” for our V4-V5 dataset. These observation matrices report counts, which are the number of reads assigned to each oligotype in each sample (Table 1). We then converted counts to percent abundances within each sample and used these normalized relative abundances for subsequent analyses. We searched the most biologically relevant representative sequence of our oligotypes using blastn version 2.2.26 to assign taxonomy for each oligotype. We kept default parameters, except ‘per. identity 100’ to have hits with 100% sequence identity reported.

### Oligotype network analysis

We performed network analysis using Gephi software, version 0.9.2^42^**,** to determine the distribution of all *Synechococcus* oligotypes from both lagoons using a force-directed graph algorithm (ForceAtlas2 in Gephi software). Every dot identifies an oligotype present in at least one sampling site, and each edge on the network connects an oligotype to one or more sampling sites.

### Clade identification

We designated a clade for each oligotype’s representative sequence by matching this latter to a key reference database of 16S rRNA gene sequences from cultured *Synechococcus*^6^. *Synechococcus* sequences downloaded from NCBI GenBank clade classifications were obtained from the following sources^4,6^. We added the representative sequences for each oligotype to the *Synechococcus* database, and we aligned them with Muscle (version 3.8.4,^17^). We used exact matches between each oligotype *Synechococcus* sequence and the *Synechococcus* sequence database to infer clade designation.

### Statistical analyses

To group oligotypes statistically, we computed a principal components analysis (PCA) using R package “ggfortify” with respect to a sample matrix of *Synechococcus* oligotype reads normalized to total *Synechococcus* reads for that sample. Each oligotype was projected onto the first two PCs of the matrix. To investigate environmental correlates of oligotype grouping, multiple regressions of each of the first two PCs were computed against the three environmental factors, which are the water temperature, salinity, and the concentration of nitrate.

## Supplementary Information

Supplementary Information.

## Data Availability

Datasets can be found at EBI under the Accession number ERX947554. The bacterial metagenome and 16S rRNA raw read sequence datasets were deposited in EMBL-EBI https://www.ebi.ac.uk/metagenomics/studies/MGYS00001977 accessed 1 December 2021 and OSD GitHub https://github.com/MicroB3-IS/osd-analysis/wiki accessed 1 December 2021.
