# Supplementary material for: Spatio-temporal patterns of Synechococcus oligotypes in Moroccan lagoonal environments
Source: Sci Rep. 2023 Jan 3;13:110. doi: 10.1038/s41598-022-27263-y (PMC9810706; doi:10.1038/s41598-022-27263-y)
Supplement: Supplementary file 1 — Supplementary Information. [file 41598_2022_27263_MOESM1_ESM.docx]

**Supplementary material**

**Supplementary Table S1. Key database for the 31 *Synechococcus*oligotypes including their accession number on NCBI, source, clade or subclade affiliations, and sequence length**

| **Oligotype ID** | **Strain name** | **Accession number** | **Description** | **Source** | **Subclade or Clade ID best match** | **Clade ID** | **Length** | **Number of bp different from the best match** |
| --- | --- | --- | --- | --- | --- | --- | --- | --- |
| O1 | WH8103 | AF311293.1 | *Synechococcus*sp. WH 8103 16S ribosomal RNA gene partial sequence | Ahlgren & Rocap 2012 | III | III | 1438 | 0 (exact match) |
| O2 | KORDI-15 | FJ497743.1 | *Synechococcus* sp. KORDI-15 16S ribosomal RNA genepartial sequence | Choi & Noh 2009 | Subcluster 5.3 | 5.3 | 1227 | 0 (exact match) |
| O3 | A15-74 | JF306716.1 | *Synechococcus* sp. A15-74 16S ribosomal RNA genepartial sequence | Mazard et al. 2011 | VII | VII | 1099 | 0 (exact match) |
| O4 | CC9902 | CP000097 | *Synechococcus* sp. CC9902 chromosomecomplete genome | Ahlgren & Rocap 2012 | IV | IV | 1479 | 0 (exact match) |
| O5 | RCC61 | JF306685.1 | *Synechococcus* sp. RCC61 16S ribosomal RNA genepartial sequence | Mazard et al. 2011 | SC 5.3 | 5.3 | 1148 | 1 |
| O6 | MV0210 | KU867926.1 | *Synechococcus* sp. MV0210 16S ribosomal RNA gene | Hunter-Cevera et al. 2016 | IE | I | 1148 | 0 (exact match) |
| O7 | UW92 | JQ421031.1 | *Synechococcus* sp. UW92 16S ribosomal RNA genepartial sequence | Ahlgren & Rocap 2012 | VII | VII | 1019 | 1 |
| O8 | MV1308 | KU867940.1 | *Synechococcus* sp. MV1308 16S ribosomal RNA gene | Hunter-Cevera et al. 2016 | IC | I | 1148 | 1 |
| O9 | MV1307 | KU867924.1 | *Synechococcus* sp. MV1307 16S ribosomal RNA gene | Hunter-Cevera et al. 2016 | IE | I | 1148 | 1 |
| O10 | MV0605E | KU867943.1 | *Synechococcus* sp. MV0605E 16S ribosomal RNA gene | Hunter-Cevera et al. 2016 | CB5 | CB5 | 1148 | 0 (exact match) |
| O11 | KORDI-15 | FJ497743.1 | *Synechococcus* sp. KORDI-15 16S ribosomal RNA genepartial sequence | Choi & Noh 2009 | Subcluster 5.3 | 5.3 | 1227 | 1 |
| O12 | CC9902 | CP000097 | *Synechococcus* sp. CC9902 chromosomecomplete genome | Ahlgren & Rocap 2012 | IV | IV | 1479 | 1 |
| O13 | KORDI-71 | FJ497742.1 | *Synechococcus* sp. KORDI-71 16S ribosomal RNA genepartial sequence | Choi & Noh 2009 | IX | IX | 1145 | 0 (exact match) |
| O14 | MV1308 | KU867940.1 | *Synechococcus* sp. MV1308 16S ribosomal RNA gene | Hunter-Cevera et al. 2016 | IC | I | 1148 | 0 (exact match) |
| O15 | WH8102 | NC_005070 | *Synechococcus* sp. WH8102 complete genome | Ahlgren & Rocap 2012 | III | III | 1464 | 2 |
| O16 | WH8016 | AY172834.1 | *Synechococcus* sp. WH 8016 16S ribosomal RNA genepartial sequence | Mazard et al. 2011 | I | I | 1439 | 1 |
| O17 | KORDI-49 | FJ497747.1 | *Synechococcus* sp. KORDI-49 16S ribosomal RNA genepartial sequence | Choi & Noh 2009 | WPC1 | WPC1 | 1380 | 1 |
| O18 | WH8101 | AF001480.1 | AF001480 *Synechococcus* WH8101 16S ribosomal RNA genepartial sequence | Ahlgren & Rocap 2012 | VIII | VIII | 1437 | 1 |
| O19 | WH8103 | AF311293.1 | *Synechococcus* sp. WH 8103 16S ribosomal RNA genepartial sequence | Ahlgren & Rocap 2012 | III | III | 1438 | 1 |
| O20 | KORDI-15 | FJ497743.1 | *Synechococcus* sp. KORDI-15 16S ribosomal RNA genepartial sequence | Choi & Noh 2009 | Subcluster 5.3 | 5.3 | 1227 | 2 |
| O21 | MV1605B | KU867931.1 | *Synechococcus* sp. MV1605B 16S ribosomal RNA gene | Hunter-Cevera et al. 2016 | III | III | 1148 | 1 |
| O22 | KORDI-15 | FJ497743.1 | *Synechococcus* sp. KORDI-15 16S ribosomal RNA genepartial sequence | Choi & Noh 2009 | Subcluster 5.3 | 5.3 | 1227 | 2 |
| O23 | WH8103 | AF311293.1 | *Synechococcus* sp. WH 8103 16S ribosomal RNA genepartial sequence | Ahlgren & Rocap 2012 | III | III | 1438 | 1 |
| O24 | WH8103 | AF311293.1 | *Synechococcus* sp. WH 8103 16S ribosomal RNA genepartial sequence | Ahlgren & Rocap 2012 | III | III | 1438 | 1 |
| O25 | WH8103 | AF311293.1 | *Synechococcus* sp. WH 8103 16S ribosomal RNA genepartial sequence | Ahlgren & Rocap 2012 | III | III | 1438 | 1 |
| O26 | WH8020 | AY172835.1 | *Synechococcus* sp. WH 8020 16S ribosomal RNA genepartial sequence | Ahlgren & Rocap 2012 | I | I | 1226 | 0 (exact match) |
| O27 | KORDI-15 | FJ497743.1 | *Synechococcus* sp. KORDI-15 16S ribosomal RNA genepartial sequence | Choi & Noh 2009 | Subcluster 5.3 | 5.3 | 1227 | 2 |
| O28 | MV0519B | KU867928.1 | *Synechococcus* sp. MV0519B 16S ribosomal RNA gene | Hunter-Cevera et al. 2016 | II | II | 1149 | 1 |
| O29 | WH8103 | AF311293.1 | *Synechococcus* sp. WH 8103 16S ribosomal RNA genepartial sequence | Ahlgren & Rocap 2012 | III | III | 1438 | 1 |
| O30 | WH8103 | AF311293.1 | *Synechococcus* sp. WH 8103 16S ribosomal RNA genepartial sequence | Ahlgren & Rocap 2012 | III | III | 1438 | 1 |
| O31 | WH8103 | AF311293.1 | *Synechococcus* sp. WH 8103 16S ribosomal RNA genepartial sequence | Ahlgren & Rocap 2012 | III | III | 1438 | 1 |

**Supplementary Table S2. V4-V5 Oligotype taxon assignment and representative sequences.**

|  |  |  |  | |  | |  |  |  |  | |
| --- | --- | --- | --- | --- | --- | --- | --- | --- | --- | --- | --- |
| **Oligotype** | **Oligotype ID** | **Oligotypename (Strain)** | | **Phylum** | | **Representative sequence** | | | | |  |
| TTAATCT | **O1** | **Synechococcus sp. WH 8103** | | **Cyanobacteria** | | **Synechococcus_**TACGGGAGTGGCAAGCGTTATCCGGAATTATTGGGCGTAAAGCGTCCGCAGGCGGCCCTTCAAGTCTGCTGTTAAAAAGTGGAGCTTAACTCCATCATGGCAGTGGAAACTGTTGGGCTTGAGTGTGGTAGGGGCAGAGGGAATTCCCGGTGTAGCGGTGAAATGCGTAGATATCGGGAAGAACACCAGTGGCGAAGGCGCTCTGCTGGGCCATCACTGACGCTCATGGACGAAAGCCAGGGGAGCGAAAGGGATTAGATACCCCTGTAGTCCTGGCCGTAAACGATGAACACTAGGTGTCGGGGGAATCGACCCCCTCGGTGTCGTAGCCAACGCGTTAAGTGTTCCGCCTGGGGAGTACGCACGCAAGTGTG | | | | |  |
| GACTCTC | **O2** | **Synechococcus sp. KORDI-15** | | **Cyanobacteria** | | **Synechococcus_**TACGGGAGTGGCAAGCGTTATCCGGAATTATTGGGCGTAAAGCGTCCGCAGGCGGCCTGACAAGTCTGCTGTTAAAGCGTGGAGCTTAACTCCATTTCAGCAGTGGAAACTGTCAGGCTTGAGTGTGGTAGGGGCAGAGGGAATTCCCGGTGTAGCGGTGAAATGCGTAGATATCGGGAAGAACACCAGTGGCGAAAGCGCTCTGCTGGGCCATCACTGACGCTCATGGACGAAAGCCAGGGGAGCGAAAGGGATTAGATACCCCTGTAGTCCTGGCCGTAAACGATGAACACTAGGTGTCGGGGGAATCGACCCCTCCGGTGTCGTAGCCAACGCGTTAAGTGTTCCGCCTGGGGAGTACGCACGCAAGTGTG | | | | |  |
| TTCATCT | **O3** | **Synechococcus sp. A15-74** | | **Cyanobacteria** | | Synechococcus_TACGGGAGTGGCAAGCGTTATCCGGAATTATTGGGCGTAAAGCGTCCGCAGGCGGCCCTTCAAGTCTGCTGTTAAAACGTGGAGCTTAACTCCATCATGGCAGTGGAAACTGTTGGGCTTGAGTGTGGTAGGGGCAGAGGGAATTCCCGGTGTAGCGGTGAAATGCGTAGATATCGGGAAGAACACCAGTGGCGAAGGCGCTCTGCTGGGCCATAACTGACGCTCATGGACGAAAGCCAGGGGAGCGAAAGGGATTAGATACCCCTGTAGTCCTGGCCGTAAACGATGAACACTAGGTGTCGGGGGAATCGACCCCCTCGGTGTCGTAGCCAACGCGTTAAGTGTTCCGCCTGGGGAG-TACGCACGCAAGTGTG | | | | |  |
| TTAAGCT | **O4** | **Synechococcus sp. CC9902** | | **Cyanobacteria** | | **Synechococcus_**TACGGGAGTGGCAAGCGTTATCCGGAATTATTGGGCGTAAAGCGTCCGCAGGCGGCCCTTCAAGTCTGCTGTTAAAAAGTGGAGCTTAACTCCATCATGGCAGTGGAAACTGAGGGGCTTGAGTGTGGTAGGGGCAGAGGGAATTCCCGGTGTAGCGGTGAAATGCGTAGATATCGGGAAGAACACCAGTGGCGAAGGCGCTCTGCTGGGCCATCACTGACGCTCATGGACGAAAGCCAGGGGAGCGAAAGGGATTAGATACCCCTGTAGTCCTGGCCGTAAACGATGAACACTAGGTGTCGGGGGAATCGACCCCCTCGGTGTCGTAGCCAACGCGTTAAGTGTTCCGCCTGGGGAGTACGCACGCAAGTGTG | | | | |  |
| GACTCCT | **O5** | **Synechococcus**  **sp. RCC61** | | **Cyanobacteria** | | **Synechococcus_**TACGGGAGTGGCAAGCGTTATCCGGAATTATTGGGCGTAAAGCGTCCGCAGGCGGCCTGACAAGTCTGCTGTTAAAGCGTGGAGCTTAACTCCATTTCAGCAGTGGAAACTGTCAGGCTTGAGTGTGGTAGGGGCAGAGGGAATTCCCGGTGTAGCGGTGAAATGCGTAGATATCGGGAAGAACACCAGTGGCGAAAGCGCTCTGCTGGGCCATCACTGACGCTCATGGACGAAAGCCAGGGGAGCGAAAGGGATTAGATACCCCTGTAGTCCTGGCCGTAAACGATGAACACTAGGTGTCGGGGGAATCGACCCCCTCGGTGTCGTAGCCAACGCGTTAAGTGTTCCGCCTGGGGAGTACGCACGCAAGTGTG | | | | |  |
| TTAATTC | **O6** | **Synechococcus sp. MV0210** | | **Cyanobacteria** | | **Synechococcus_**TACGGGAGTGGCAAGCGTTATCCGGAATTATTGGGCGTAAAGCGTCCGCAGGCGGCCCTTCAAGTCTGCTGTTAAAAAGTGGAGCTTAACTCCATCATGGCAGTGGAAACTGTTGGGCTTGAGTGTGGTAGGGGCAGAGGGAATTCCCGGTGTAGCGGTGAAATGCGTAGATATCGGGAAGAACACCAGTGGCGAAGGCGCTCTGCTGGGCCATCACTGACGCTCATGGACGAAAGCCAGGGGAGCGAAAGGGATTAGATACCCCTGTAGTCCTGGCCGTAAACGATGAACACTAGGTGTCGGGGGAATCGACCCCTCCGGTGTCGTAGCCAACGCGTTAAGTGTTCCGCCTGGGGAG-TACGCACGCAAGTGTG | | | | |  |
| TTCATTC | **O7** | **Synechococcus sp. UW92** | | **Cyanobacteria** | | **Synechococcus_**TACGGGAGTGGCAAGCGTTATCCGGAATTATTGGGCGTAAAGCGTCCGCAGGCGGCCCTTCAAGTCTGCTGTTAAAACGTGGAGCTTAACTCCATCATGGCAGTGGAAACTGTTGGGCTTGAGTGTGGTAGGGGCAGAGGGAATTCCCGGTGTAGCGGTGAAATGCGTAGATATCGGGAAGAACACCAGTGGCGAAGGCGCTCTGCTGGGCCATAACTGACGCTCATGGACGAAAGCCAGGGGAGCGAAAGGGATTAGATACCCCTGTAGTCCTGGCCGTAAACGATGAACACTAGGTGTCGGGGGAATCGACCCCTCCGGTGTCGTAGCCAACGCGTTAAGTGTTCCGCCTGGGGAGTACGCACGCAAGTGTG | | | | |  |
| ATAATTC | **O8** | **Synechococcus sp. MV1308** | | **Cyanobacteria** | | **Synechococcus_**TACGGGAGTGGCAAGCGTTATCCGGAATTATTGGGCGTAAAGCGTCCGCAGGCGGCCCATCAAGTCTGCTGTTAAAAAGTGGAGCTTAACTCCATCATGGCAGTGGAAACTGTTGGGCTAGAGTGTGGTAGGGGCAGAGGGAATTCCCGGTGTAGCGGTGAAATGCGTAGATATCGGGAAGAACACCAGTGGCGAAGGCGCTCTGCTGGGCCATAACTGACGCTCATGGACGAAAGCCAGGGGAGCGAAAGGGATTAGATACCCCTGTAGTCCTGGCCGTAAACGATGAACACTAGGTGTCGGGGGAATCGACCCCTCCGGTGTCGTAGCCAACGCGTTAAGTGTTCCGCCTGGGGAGTACGCACGCAAGTGTG | | | | |  |
| TTAATTT | **O9** | **Synechococcus sp. MV1307** | | **Cyanobacteria** | | **Synechococcus_**TACGGGAGTGGCAAGCGTTATCCGGAATTATTGGGCGTAAAGCGTCCGCAGGCGGCCCTTCAAGTCTGCTGTTAAAAAGTGGAGCTTAACTCCATCATGGCAGTGGAAACTGTTGGGCTTGAGTGTGGTAGGGGCAGAGGGAATTCCCGGTGTAGCGGTGAAATGCGTAGATATCGGGAAGAACACCAGTGGCGAAGGCGCTCTGCTGGGCCATCACTGACGCTCATGGACGAAAGCCAGGGGAGCGAAAGGGATTAGATACCCCTGTAGTCCTGGCCGTAAACGATGAACACTAGGTGTCGGGGGAATCGACCCCTTCGGTGTCGTAGCCAACGCGTTAAGTGTTCCGCCTGGGGAGTACGCACGCAAGTGTG | | | | |  |
| GTCTCCT | **O10** | **Synechococcus sp. MV0605E** | | **Cyanobacteria** | | **Synechococcus_**TACGGGAGTGGCAAGCGTTATCCGGAATTATTGGGCGTAAAGCGTCCGCAGGCGGTCTGTCAAGTCTGCTGTTAAAGCGTGGAGCTTAACTCCATTTCGGCAGTGGAAACTGACAGACTAGAGTGTGGTAGGGGCAGAGGGAATTCCCGGTGTAGCGGTGAAATGCGTAGATATCGGGAAGAACACCAGTGGCGAAGGCGCTCTGCTGGGCCATAACTGACGCTCATGGACGAAAGCCAGGGGAGCGAAAGGGATTAGATACCCCTGTAGTCCTGGCCGTAAACGATGAACACTAGGTGTCGGGGGAATCGACCCCCTCGGTGTCGTAGCCAACGCGTTAAGTGTTCCGCCTGGGGAGTACGCACGCAAGTGTG | | | | |  |
| TTCTCTC | **O11** | **Synechococcus sp. KORDI-15** | | **Cyanobacteria** | | **Synechococcus_**TACGGGAGTGGCAAGCGTTATCCGGAATTATTGGGCGTAAAGCGTCCGCAGGCGGCCCTTCAAGTCTGCTGTTAAAGCGTGGAGCTTAACTCCATTTCAGCAGTGGAAACTGTCAGGCTTGAGTGTGGTAGGGGCAGAGGGAATTCCCGGTGTAGCGGTGAAATGCGTAGATATCGGGAAGAACACCAGTGGCGAAAGCGCTCTGCTGGGCCATCACTGACGCTCATGGACGAAAGCCAGGGGAGCGAAAGGGATTAGATACCCCTGTAGTCCTGGCCGTAAACGATGAACACTAGGTGTCGGGGGAATCGACCCCTCCGGTGTCGTAGCCAACGCGTTAAGTGTTCCGCCTGGGGAGTACGCACGCAAGTGTG | | | | |  |
| TTAAGTC | **O12** | **Synechococcus sp. CC9902** | | **Cyanobacteria** | | **Synechococcus_**TACGGGAGTGGCAAGCGTTATCCGGAATTATTGGGCGTAAAGCGTCCGCAGGCGGCCCTTCAAGTCTGCTGTTAAAAAGTGGAGCTTAACTCCATCATGGCAGTGGAAACTGAGGGGCTTGAGTGTGGTAGGGGCAGAGGGAATTCCCGGTGTAGCGGTGAAATGCGTAGATATCGGGAAGAACACCAGTGGCGAAGGCGCTCTGCTGGGCCATCACTGACGCTCATGGACGAAAGCCAGGGGAGCGAAAGGGATTAGATACCCCTGTAGTCCTGGCCGTAAACGATGAACACTAGGTGTCGGGGGAATCGACCCCTCCGGTGTCGTAGCCAACGCGTTAAGTGTTCCGCCTGGGGAGTACGCACGCAAGTGTG | | | | |  |
| AGAATTC | **O13** | **Synechococcus sp. KORDI-71** | | **Cyanobacteria** | | **Synechococcus_**TACGGGAGTGGCAAGCGTTATCCGGAATTATTGGGCGTAAAGCGTCCGCAGGCGGTCCAGAAAGTCTGCTGTTAAAAAGTGGAGCTTAACTCCATCATGGCAGTGGAAACTTCTGGACTAGAGTGTGGTAGGGGCAGAGGGAATTCCCGGTGTAGCGGTGAAATGCGTAGATATCGGGAAGAACACCAGTGGCGAAGGCGCTCTGCTGGGCCATAACTGACGCTCATGGACGAAAGCCAGGGGAGCGAAAGGGATTAGATACCCCTGTAGTCCTGGCCGTAAACGATGAACACTAGGTGTCGGGGGAATCGACCCCTCCGGTGTCGTAGCCAACGCGTTAAGTGTTCCGCCTGGGGAGTACGCACGCAAGTGTG | | | | |  |
| AAAATTC | **O14** | **Synechococcus sp. MV1308** | | **Cyanobacteria** | | **Synechococcus_**TACGGGAGTGGCAAGCGTTATCCGGAATTATTGGGCGTAAAGCGTCCGCAGGCGGCCCAACAAGTCTGCTGTTAAAAAGTGGAGCTTAACTCCATCATGGCAGTGGAAACTGTTGGGCTAGAGTGTGGTAGGGGCAGAGGGAATTCCCGGTGTAGCGGTGAAATGCGTAGATATCGGGAAGAACACCAGTGGCGAAGGCGCTCTGCTGGGCCATCACTGACGCTCATGGACGAAAGCCAGGGGAGCGAAAGGGATTAGATACCCCTGTAGTCCTGGCCGTAAACGATGAACACTAGGTGTCGGGGGAATCGACCCCTCCGGTGTCGTAGCCAACGCGTTAAGTGTTCCGCCTGGGGAGTACGCACGCAAGTGTG | | | | |  |
| GACATCT | **O15** | **Synechococcus sp. 8102** | | **Cyanobacteria** | | **Synechococcus_**TACGGGAGTGGCAAGCGTTATCCGGAATTATTGGGCGTAAAGCGTCCGCAGGCGGCCTGACAAGTCTGCTGTTAAAGCGTGGAGCTTAACTCCATCATGGCAGTGGAAACTGTTGGGCTTGAGTGTGGTAGGGGCAGAGGGAATTCCCGGTGTAGCGGTGAAATGCGTAGATATCGGGAAGAACACCAGTGGCGAAGGCGCTCTGCTGGGCCATCACTGACGCTCATGGACGAAAGCCAGGGGAGCGAAAGGGATTAGATACCCCTGTAGTCCTGGCCGTAAACGATGAACACTAGGTGTCGGGGGAATCGACCCCCTCGGTGTCGTAGCCAACGCGTTAAGTGTTCCGCCTGGGGAGTACGCACGCAAGTGTG | | | | |  |
| TTAACTC | **O16** | **Synechococcus sp. WH 8016** | | **Cyanobacteria** | | **Synechococcus_**TACGGGAGTGGCAAGCGTTATCCGGAATTATTGGGCGTAAAGCGTCCGCAGGCGGCCCTTCAAGTCTGCTGTTAAAAAGTGGAGCTTAACTCCATCATGGCAGTGGAAACTGTCAGGCTTGAGTGTGGTAGGGGCAGAGGGAATTCCCGGTGTAGCGGTGAAATGCGTAGATATCGGGAAGAACACCAGTGGCGAAAGCGCTCTGCTGGGCCATCACTGACGCTCATGGACGAAAGCCAGGGGAGCGAAAGGGATTAGATACCCCTGTAGTCCTGGCCGTAAACGATGAACACTAGGTGTCGGGGGAATCGACCCCTCCGGTGTCGTAGCCAACGCGTTAAGTGTTCCGCCTGGGGAGTACGCACGCAAGTGTG | | | | |  |
| AGCATCT | **O17** | **Synechococcus sp. KORDI-49** | | **Cyanobacteria** | | **Synechococcus_**TACGGGAGTGGCAAGCGTTATCCGGAATTATTGGGCGTAAAGCGTCCGCAGGCGGCCCAGAAAGTCTGCTGTTAAAACGTGGAGCTTAACTCCATCATGGCAGTGGAAACTGTTGGGCTTGAGTGTGGTAGGGGCAGAGGGAATTCCCGGTGTAGCGGTGAAATGCGTAGATATCGGGAAGAACACCAGTGGCGAAGGCGCTCTGCTGGGCCATCACTGACGCTCATGGACGAAAGCCAGGGGAGCGAAAGGGATTAGATACCCCTGTAGTCCTGGCCGTAAACGATGAACACTAGGTGTCGGGGGAATCGACCCCCTCGGTGTCGTAGCCAACGCGTTAAGTGTTCCGCCTGGGGAG-TACGCACGCAAGTGTG | | | | |  |
| ATAATCT | **O18** | **Synechococcus sp. WH8101** | | **Cyanobacteria** | | **Synechococcus_**TACGGGAGTGGCAAGCGTTATCCGGAATTATTGGGCGTAAAGCGTCCGCAGGCGGCCCATCAAGTCTGCTGTTAAAAAGTGGAGCTTAACTCCATCATGGCAGTGGAAACTGTTGGGCTAGAGTGTGGTAGGGGCAGAGGGAATTCCCGGTGTAGCGGTGAAATGCGTAGATATCGGGAAGAACACCAGTGGCGAAGGCGCTCTGCTGGGCCATAACTGACGCTCATGGACGAAAGCCAGGGGAGCGAAAGGGATTAGATACCCCTGTAGTCCTGGCCGTAAACGATGAACACTAGGTGTCGGGGGAATCGACCCCCTCGGTGTCGTAGCCAACGCGTTAAGTGTTCCGCCTGGGGAGTACGCACGCAAGTGTG | | | | |  |
| TTAACCT | **O19** | **Synechococcus sp. WH 8103** | | **Cyanobacteria** | | **Synechococcus_**TACGGGAGTGGCAAGCGTTATCCGGAATTATTGGGCGTAAAGCGTCCGCAGGCGGCCCTTCAAGTCTGCTGTTAAAAAGTGGAGCTTAACTCCATCATGGCAGTGGAAACTGTCGGGCTTGAGTGTGGTAGGGGCAGAGGGAATTCCCGGTGTAGCGGTGAAATGCGTAGATATCGGGAAGAACACCAGTGGCGAAGGCGCTCTGCTGGGCCATCACTGACGCTCATGGACGAAAGCCAGGGGAGCGAAAGGGATTAGATACCCCTGTAGTCCTGGCCGTAAACGATGAACACTAGGTGTCGGGGGAATCGACCCCCTCGGTGTCGTAGCCAACGCGTTAAGTGTTCCGCCTGGGGAGTACGCACGCAAGTGTG | | | | |  |
| TTATCTC | **O20** | **Synechococcus sp. KORDI-15** | | **Cyanobacteria** | | **Synechococcus_**TACGGGAGTGGCAAGCGTTATCCGGAATTATTGGGCGTAAAGCGTCCGCAGGCGGCCCTTCAAGTCTGCTGTTAAAAAGTGGAGCTTAACTCCATTTCAGCAGTGGAAACTGTCAGGCTTGAGTGTGGTAGGGGCAGAGGGAATTCCCGGTGTAGCGGTGAAATGCGTAGATATCGGGAAGAACACCAGTGGCGAAAGCGCTCTGCTGGGCCATCACTGACGCTCATGGACGAAAGCCAGGGGAGCGAAAGGGATTAGATACCCCTGTAGTCCTGGCCGTAAACGATGAACACTAGGTGTCGGGGGAATCGACCCCTCCGGTGTCGTAGCCAACGCGTTAAGTGTTCCGCCTGGGGAGTACGCACGCAAGTGTG | | | | |  |
| TAAATCT | **O21** | **Synechococcus sp. MV1605B** | | **Cyanobacteria** | | **Synechococcus_**TACGGGAGTGGCAAGCGTTATCCGGAATTATTGGGCGTAAAGCGTCCGCAGGCGGCCCTACAAGTCTGCTGTTAAAAAGTGGAGCTTAACTCCATCATGGCAGTGGAAACTGTTGGGCTTGAGTGTGGTAGGGGCAGAGGGAATTCCCGGTGTAGCGGTGAAATGCGTAGATATCGGGAAGAACACCAGTGGCGAAGGCGCTCTGCTGGGCCATCACTGACGCTCATGGACGAAAGCCAGGGGAGCGAAAGGGATTAGATACCCCTGTAGTCCTGGCCGTAAACGATGAACACTAGGTGTCGGGGGAATCGACCCCCTCGGTGTCGTAGCCAACGCGTTAAGTGTTCCGCCTGGGGAGTACGCACGCAAGTGTG | | | | |  |
| TTCTCCT | **O22** | **Synechococcus sp. KORDI-15** | | **Cyanobacteria** | | **Synechococcus_**TACGGGAGTGGCAAGCGTTATCCGGAATTATTGGGCGTAAAGCGTCCGCAGGCGGCCCTTCAAGTCTGCTGTTAAAGCGTGGAGCTTAACTCCATTTCAGCAGTGGAAACTGTCAGGCTTGAGTGTGGTAGGGGCAGAGGGAATTCCCGGTGTAGCGGTGAAATGCGTAGATATCGGGAAGAACACCAGTGGCGAAAGCGCTCTGCTGGGCCATCACTGACGCTCATGGACGAAAGCCAGGGGAGCGAAAGGGATTAGATACCCCTGTAGTCCTGGCCGTAAACGATGAACACTAGGTGTCGGGGGAATCGACCCCCTCGGTGTCGTAGCCAACGCGTTAAGTGTTCCGCCTGGGGAGTACGCACGCAAGTGTG | | | | |  |
| TTACTCT | **O23** | **Synechococcus sp. WH 8103** | | **Cyanobacteria** | | **Synechococcus_**TACGGGAGTGGCAAGCGTTATCCGGAATTATTGGGCGTAAAGCGTCCGCAGGCGGCCCTTCAAGTCTGCTGTTAAAAAGTGGAGCTTAACTCCATCCTGGCAGTGGAAACTGTTGGGCTTGAGTGTGGTAGGGGCAGAGGGAATTCCCGGTGTAGCGGTGAAATGCGTAGATATCGGGAAGAACACCAGTGGCGAAGGCGCTCTGCTGGGCCATCACTGACGCTCATGGACGAAAGCCAGGGGAGCGAAAGGGATTAGATACCCCTGTAGTCCTGGCCGTAAACGATGAACACTAGGTGTCGGGGGAATCGACCCCCTCGGTGTCGTAGCCAACGCGTTAAGTGTTCCGCCTGGGGAGTACGCACGCAAGTGTG | | | | |  |
| TTGATCT | **O24** | **Synechococcus sp. WH 8103** | | **Cyanobacteria** | | **Synechococcus_**TACGGGAGTGGCAAGCGTTATCCGGAATTATTGGGCGTAAAGCGTCCGCAGGCGGCCCTTCAAGTCTGCTGTTAAAAGGTGGAGCTTAACTCCATCATGGCAGTGGAAACTGTTGGGCTTGAGTGTGGTAGGGGCAGAGGGAATTCCCGGTGTAGCGGTGAAATGCGTAGATATCGGGAAGAACACCAGTGGCGAAGGCGCTCTGCTGGGCCATCACTGACGCTCATGGACGAAAGCCAGGGGAGCGAAAGGGATTAGATACCCCTGTAGTCCTGGCCGTAAACGATGAACACTAGGTGTCGGGGGAATCGACCCCCTCGGTGTCGTAGCCAACGCGTTAAGTGTTCCGCCTGGGGAGTACGCACGCAAGTGTG | | | | |  |
| TTAGTCT | **O25** | **Synechococcus sp. WH 8103** | | **Cyanobacteria** | | **Synechococcus_**TACGGGAGTGGCAAGCGTTATCCGGAATTATTGGGCGTAAAGCGTCCGCAGGCGGCCCTTCAAGTCTGCTGTTAAAAAGTGGAGCTTAACTCCATCGTGGCAGTGGAAACTGTTGGGCTTGAGTGTGGTAGGGGCAGAGGGAATTCCCGGTGTAGCGGTGAAATGCGTAGATATCGGGAAGAACACCAGTGGCGAAGGCGCTCTGCTGGGCCATCACTGACGCTCATGGACGAAAGCCAGGGGAGCGAAAGGGATTAGATACCCCTGTAGTCCTGGCCGTAAACGATGAACACTAGGTGTCGGGGGAATCGACCCCCTCGGTGTCGTAGCCAACGCGTTAAGTGTTCCGCCTGGGGAGTACGCACGCAAGTGTG | | | | |  |
| TAAATTC | **O26** | **Synechococcus sp. WH 8020** | | **Cyanobacteria** | | **Synechococcus_**TACGGGAGTGGCAAGCGTTATCCGGAATTATTGGGCGTAAAGCGTCCGCAGGCGGCCCTACAAGTCTGCTGTTAAAAAGTGGAGCTTAACTCCATCATGGCAGTGGAAACTGTTGGGCTTGAGTGTGGTAGGGGCAGAGGGAATTCCCGGTGTAGCGGTGAAATGCGTAGATATCGGGAAGAACACCAGTGGCGAAGGCGCTCTGCTGGGCCATCACTGACGCTCATGGACGAAAGCCAGGGGAGCGAAAGGGATTAGATACCCCTGTAGTCCTGGCCGTAAACGATGAACACTAGGTGTCGGGGGAATCGACCCCTCCGGTGTCGTAGCCAACGCGTTAAGTGTTCCGCCTGGGGAGTACGCACGCAAGTGTG | | | | |  |
| GACTTCT | **O27** | **Synechococcus sp. KORDI-15** | | **Cyanobacteria** | | **Synechococcus_**TACGGGAGTGGCAAGCGTTATCCGGAATTATTGGGCGTAAAGCGTCCGCAGGCGGCCTGACAAGTCTGCTGTTAAAGCGTGGAGCTTAACTCCATTTCAGCAGTGGAAACTGTTGGGCTTGAGTGTGGTAGGGGCAGAGGGAATTCCCGGTGTAGCGGTGAAATGCGTAGATATCGGGAAGAACACCAGTGGCGAAGGCGCTCTGCTGGGCCATCACTGACGCTCATGGACGAAAGCCAGGGGAGCGAAAGGGATTAGATACCCCTGTAGTCCTGGCCGTAAACGATGAACACTAGGTGTCGGGGGAATCGACCCCCTCGGTGTCGTAGCCAACGCGTTAAGTGTTCCGCCTGGGGAGTACGCACGCAAGTGTG | | | | |  |
| TTAATCC | **O28** | **Synechococcus sp. MV0519B** | | **Cyanobacteria** | | **Synechococcus_**TACGGGAGTGGCAAGCGTTATCCGGAATTATTGGGCGTAAAGCGTCCGCAGGCGGCCCTTCAAGTCTGCTGTTAAAAAGTGGAGCTTAACTCCATCATGGCAGTGGAAACTGTTGGGCTTGAGTGTGGTAGGGGCAGAGGGAATTCCCGGTGTAGCGGTGAAATGCGTAGATATCGGGAAGAACACCAGTGGCGAAGGCGCTCTGCTGGGCCATCACTGACGCTCATGGACGAAAGCCAGGGGAGCGAAAGGGATTAGATACCCCTGTAGTCCTGGCCGTAAACGATGAACACTAGGTGTCGGGGGAATCGACCCCCCCGGTGTCGTAGCCAACGCGTTAAGTGTTCCGCCTGGGGAGTACGCACGCAAGTGTG | | | | |  |
| TCAATCT | **O29** | **Synechococcus sp. WH 8103** | | **Cyanobacteria** | | **Synechococcus_**TACGGGAGTGGCAAGCGTTATCCGGAATTATTGGGCGTAAAGCGTCCGCAGGCGGCCCTCCAAGTCTGCTGTTAAAAAGTGGAGCTTAACTCCATCATGGCAGTGGAAACTGTTGGGCTTGAGTGTGGTAGGGGCAGAGGGAATTCCCGGTGTAGCGGTGAAATGCGTAGATATCGGGAAGAACACCAGTGGCGAAGGCGCTCTGCTGGGCCATCACTGACGCTCATGGACGAAAGCCAGGGGAGCGAAAGGGATTAGATACCCCTGTAGTCCTGGCCGTAAACGATGAACACTAGGTGTCGGGGGAATCGACCCCCTCGGTGTCGTAGCCAACGCGTTAAGTGTTCCGCCTGGGGAGTACGCACGCAAGTGTG | | | | |  |
| GAAATCT | **O30** | **Synechococcus sp. WH 8103** | | **Cyanobacteria** | | **Synechococcus_**TACGGGAGTGGCAAGCGTTATCCGGAATTATTGGGCGTAAAGCGTCCGCAGGCGGCCTGACAAGTCTGCTGTTAAAAAGTGGAGCTTAACTCCATCATGGCAGTGGAAACTGTTGGGCTTGAGTGTGGTAGGGGCAGAGGGAATTCCCGGTGTAGCGGTGAAATGCGTAGATATCGGGAAGAACACCAGTGGCGAAGGCGCTCTGCTGGGCCATCACTGACGCTCATGGACGAAAGCCAGGGGAGCGAAAGGGATTAGATACCCCTGTAGTCCTGGCCGTAAACGATGAACACTAGGTGTCGGGGGAATCGACCCCCTCGGTGTCGTAGCCAACGCGTTAAGTGTTCCGCCTGGGGAGTACGCACGCAAGTGTG | | | | |  |
| CTAATCT | **O31** | **Synechococcus sp. WH 8103** | | **Cyanobacteria** | | **Synechococcus_**TACGGGAGTGGCAAGCGTTATCCGGAATTATTGGGCGTAAAGCGTCCGCAGGCGGCCCCTCAAGTCTGCTGTTAAAAAGTGGAGCTTAACTCCATCATGGCAGTGGAAACTGTTGGGCTTGAGTGTGGTAGGGGCAGAGGGAATTCCCGGTGTAGCGGTGAAATGCGTAGATATCGGGAAGAACACCAGTGGCGAAGGCGCTCTGCTGGGCCATCACTGACGCTCATGGACGAAAGCCAGGGGAGCGAAAGGGATTAGATACCCCTGTAGTCCTGGCCGTAAACGATGAACACTAGGTGTCGGGGGAATCGACCCCCTCGGTGTCGTAGCCAACGCGTTAAGTGTTCCGCCTGGGGAGTACGCACGCAAGTGTG | | | | |  |
